# Periodontal Architecture in Ectodermal Dysplasia: An Observational Clinical and Histological Study

**DOI:** 10.1111/odi.70196

**Published:** 2026-01-08

**Authors:** Marco Montevecchi, Margherita Giorgia Liguori, Ilaria Santini, Nicola Marco Sforza, Giulia Querzoli, Leoluca Valeriani

**Affiliations:** ^1^ Division of Periodontology and Implantology, Department of Biomedical and Neuromotor Sciences, School of Dentistry Alma Mater Studiorum – University of Bologna Bologna Italy; ^2^ Department of Life, Health and Environmental Sciences University of L'Aquila L'Aquila Italy; ^3^ Private Practice Bologna Italy; ^4^ Section of Anatomic Pathology S. Orsola Hospital IRCCS Azienda Ospedaliero Universitaria di Bologna Bologna Italy; ^5^ Department of Medical and Surgical Sciences (DIMEC), Alma Mater Studiorum Università di Bologna Bologna Italy; ^6^ e‐Campus University Novedrate Como Italy

**Keywords:** ectodermal dysplasia, gingival phenotype, histology, keratinized mucosa, periodontal anatomy, soft tissue thickness

## Abstract

**Objective:**

To investigate gingival and periodontal characteristics in Ectodermal dysplasia (ED), focusing on soft‐tissue phenotype, anatomical variations, and periodontal architecture.

**Materials and Methods:**

Observational clinical study of 11 individuals (16–30 years) with confirmed clinical or genetic ED diagnosis. Only periodontally healthy patients were included. Assessments comprised Plaque Index and modified Gingival Index, Probing Depth (PD), Bleeding on Probing (BoP), Gingival Phenotype, Widths of Attached Gingiva (AGW) and Keratinized Mucosa (KMW). A histological sample of keratinized gingiva was obtained from a single patient to complement the clinical evaluation.

**Results:**

A consistently thin gingival phenotype was found, with medium/thick tissues confined to isolated molars. AGW and KMW were within ranges but unevenly distributed; several mandibular areas showed reduced or absent attached gingiva. Some sites with minimal AGW lacked recession, suggesting a congenital condition. Gingival fragility and translucency complicated standard indices, potentially overestimating PD and BoP. Histology showed epithelial projections into the submucosa and poorly organized connective tissue with loosely arranged collagen and loss of normal supracrestal orientation. Scattered epithelial nests and ectopic enamel matrix deposits are visible within the submucosa.

**Conclusions:**

Individuals with ED may exhibit soft‐tissue features and periodontal architecture distinct from typical patterns, supporting the need for tailored clinical assessment and potentially modified diagnostic criteria.

## 1 Introduction

Ectodermal dysplasia (ED) refers to a broad and heterogeneous group of rare congenital disorders characterized by the abnormal development (hypoplasia, aplasia, or dysplasia) of two or more ectoderm‐derived structures, including hair, teeth, nails, skin, and sweat glands (Priolo 2009). First described by Danz in 1792 and formally named by Weech in 1929, ED now encompasses more than 200 subtypes, reflecting the intricate embryological and genetic interactions across germ layers (Deshmukh and Prashanth 2012; Pinheiro and Freire‐Maia 1994). The most prevalent form is X‐linked hypohidrotic ectodermal dysplasia (XLHED), or Christ‐Siemens‐Touraine syndrome (OMIM 305100), caused by mutations in the EDA gene and representing approximately 70% of ED cases (Anbouba et al. 2020; Kupietzky and Houpt 1995). The estimated prevalence of hypohidrotic forms ranges from 1 in 5000 to 1 in 10,000 live births, with XLHED affecting about 1 in 50,000–100,000 males. Recent reports suggest approximately 7000 documented cases worldwide (Wohlfart et al. 2020; Guckes et al. 1991).

**FIGURE 1:**
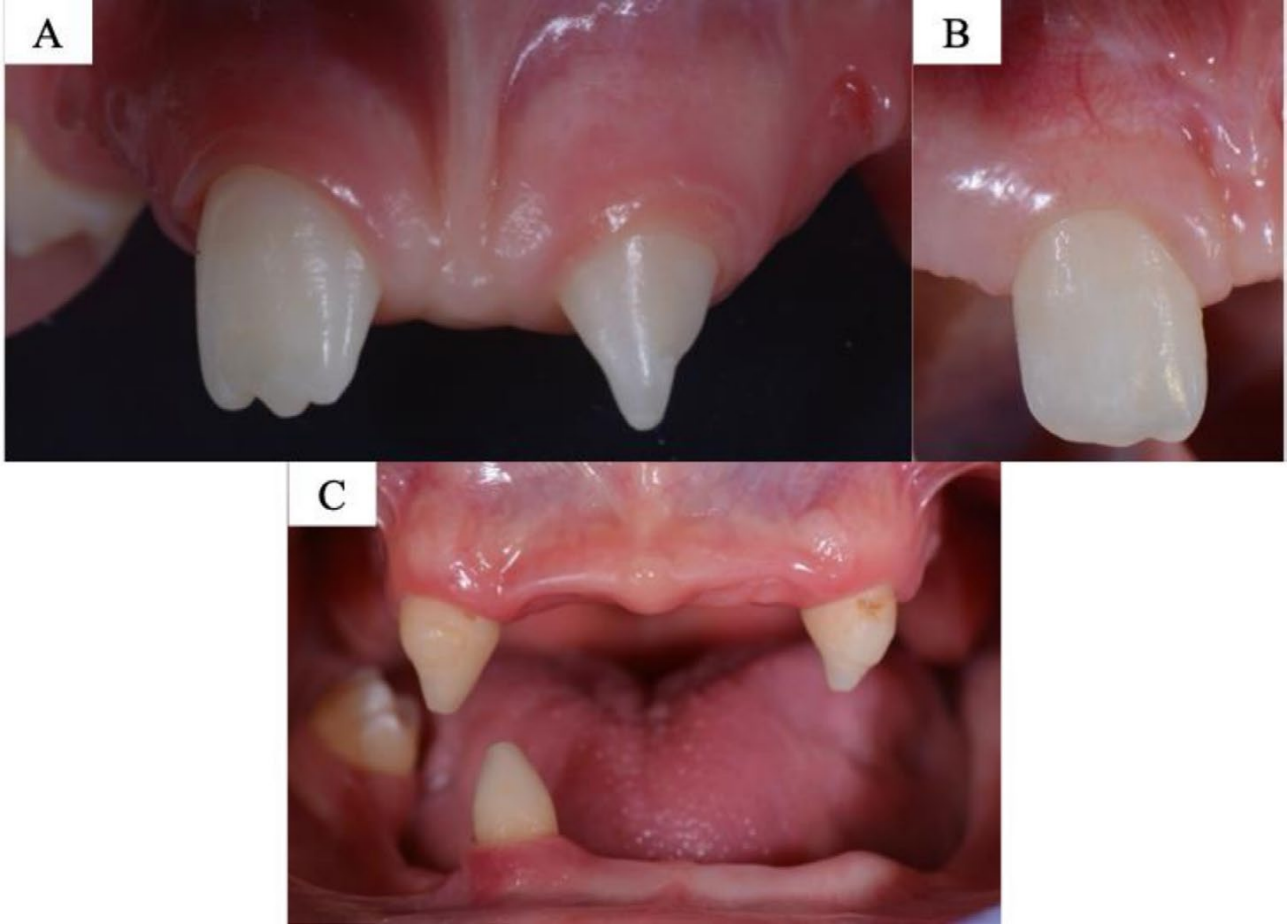
(A) Clinical image of the second sextant in a 16‐year‐old male with ectodermal dysplasia. Typical conical and peg‐shaped anterior teeth with reduced mesio‐distal width are observable. It is possible to read also the severely reduced alveolar process of the edentulous ridges. (B) Focus on the marginal periodontium of a 17‐year‐old female, where gingival tissue reveals the underlying capillary network. (C) Intraoral view of a 17‐year‐old male showing oligodontia, absence of the oral vestibulum, and a severely reduced alveolar process in the lower arch, which presents with a thin, blade‐like morphology.

Both males and female carriers may exhibit significant oro‐facial anomalies with functional, psychological, and esthetic consequences (Hickey and Vergo 2001). These include variations in tooth number (anodontia, hypodontia, or oligodontia), morphology, and structure, as well as altered eruption timing and persistence of deciduous teeth, especially molars and canines (Rad et al. 2007). Enamel defects, ranging from hypoplasia to hypo‐mineralization, further compromise crown integrity. Hypodontia, observed in the majority of individuals with XLHED, typically involves the permanent canines and first molars, and is often accompanied by anomalies in tooth size and shape, distinguishing these patients from the general population (Hickey and Vergo 2001; Kearns et al. 1999; Kramer et al. 2007). Teeth, when present, are frequently reduced in size and may display a characteristic conical or peg‐shaped appearance (Figure 1) (Kearns et al. 1999). The generalized absence of teeth leads to underdevelopment of the alveolar processes, with impaired vertical and horizontal bone growth (Figure 1). These alterations have a profound impact on various aspects of daily life, including masticatory function, phonetics, social interaction, and psychological well‐being (Bergendal et al. 1991).

Given that the most prominent clinical challenges in ED involve the absence of teeth, in recent years, the literature has largely prioritized prosthetic and implant‐supported rehabilitation of individuals with ED to compensate for extensive tooth loss (Anbouba et al. 2020; Kirmeier et al. 2009; Zou et al. 2014; Clauss et al. 2014; Goker et al. 2020). While these efforts have advanced the field of prosthetic and surgical dentistry, the status of periodontal tissues in individuals with ED has received comparatively limited attention. As a result, evidence regarding periodontal parameters, soft tissue characteristics, and susceptibility to periodontal disease in this population remains limited. To date, no clinical studies have assessed whether the distinctive dental and skeletal features of ED are associated with alterations in periodontal anatomy or function. This lack of data underscores the need for focused investigations on periodontal health in individuals with ED, which may hold clinical significance for treatment planning and long‐term outcomes.

The present study is an observational clinical investigation aimed at evaluating the macroscopic anatomical and functional characteristics of the superficial periodontium in individuals affected by ED. This clinical assessment is complemented by a histological analysis of keratinized gingiva in an ED subject.

## 2 Materials and Methods

### 2.1 Study Setting and Ethical Approval

Participants were consecutively recruited between December 2020 and July 2021 at the Dental Care Service for Disabled Individuals in Developmental Age, Department of Biomedical and Neuromotor Sciences (DIBINEM), Alma Mater Studiorum, University of Bologna, with the support of the National Ectodermal Dysplasia Association (A.N.D.E.). All participants, together with their parents or legal representatives (in case of minors), provided written informed consent and completed a paper‐based demographic and medical history questionnaire. This observational descriptive clinical study was approved by the Independent Ethics Committee of the University Hospital of Bologna, S. Orsola‐Malpighi Polyclinic (approval number 27/2014/O/Oss).

### 2.2 Inclusion and Exclusion Criteria

Individuals with a clinical and/or genetic diagnosis of ectodermal dysplasia, aged between 16 and 30 years and presenting at least one fully erupted tooth, were eligible for inclusion. Exclusion criteria included the regular use of medications and/or the presence of local or systemic conditions (other than ED) that could affect the periodontal tissues and smoking habits. The presence of fixed orthodontic appliances or fixed prostheses was considered a limiting factor but not an exclusion criterion for the involved sites due to the high prevalence of such devices among ED patients, even at a young age.

Only periodontally healthy individuals were included in the study, based on the current classification of periodontal and peri‐implant diseases and conditions (Papapanou et al. 2018). Patients diagnosed with gingivitis received nonsurgical periodontal treatment and were re‐evaluated after 2 weeks. Only those who had achieved periodontal health at that time were included in the final sample. Patients who failed to reach periodontal health or were diagnosed with periodontitis were excluded from the study.

### 2.3 Clinical Procedures

**FIGURE 2:**
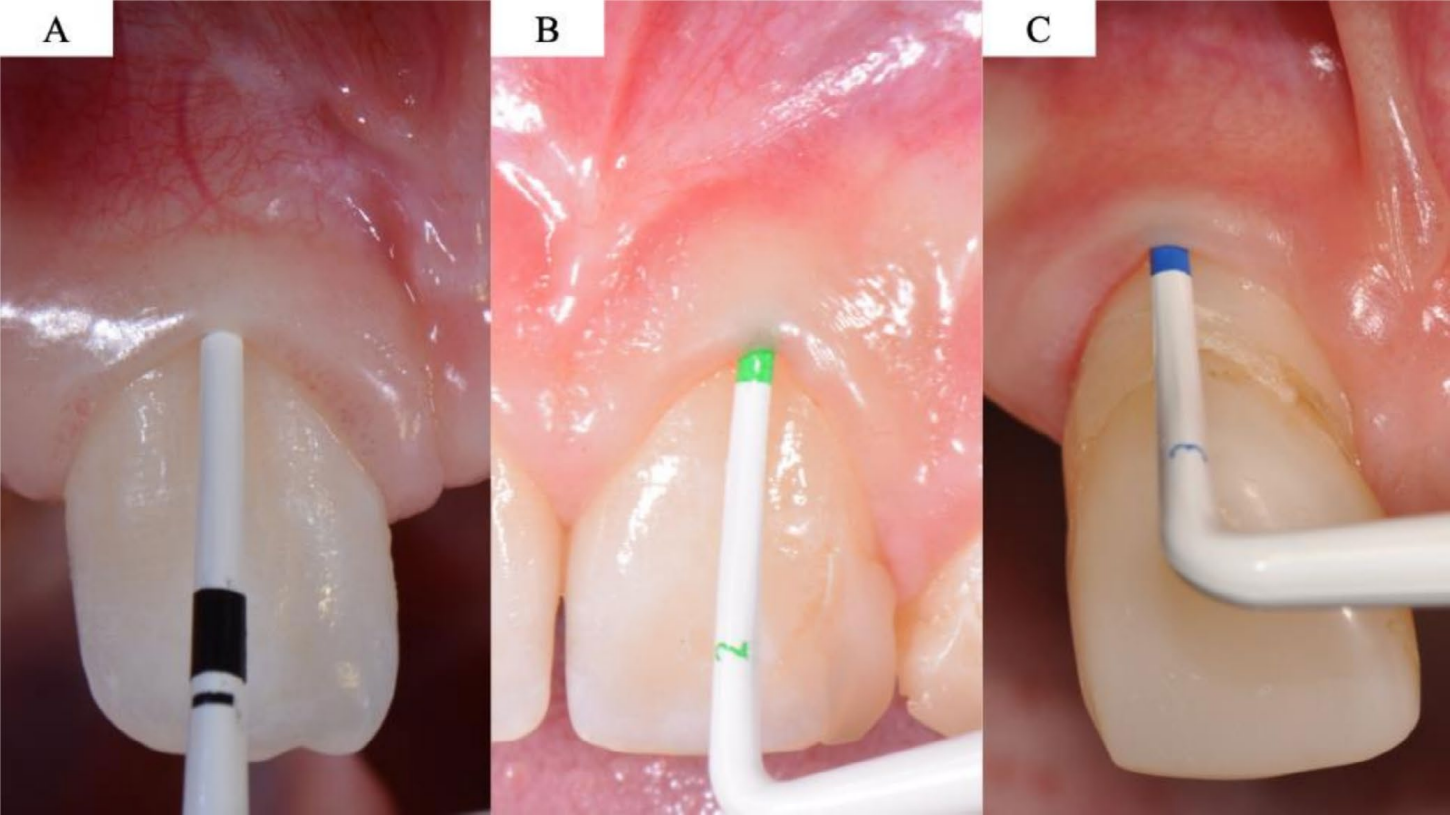
Gingival phenotype assessment using the Colorvue Biotype probe on ectodermal dysplasia subjects. (A) Thin phenotype: The white band is visible through the gingiva. (B) Medium phenotype: The green band is visible when the white is not. (C) Thick or very thick phenotype: The blue band is used when neither the white nor green bands can be seen through the tissue.

All clinical assessments were performed by the same calibrated operator to ensure methodological consistency. Gingivitis treatment was performed during the same session and included debridement using manual and/or mechanical instruments, tailored to the individual needs, along with selective polishing using a silicone rubber cup and prophylactic paste of variable grit. Any plaque or calculus deposits on removable prostheses were also removed. Instruction and motivation were provided to ensure proper oral hygiene for both teeth and prostheses. A clinical intraoral examination was performed using a UNC‐15 periodontal probe (Hu‐Friedy, Chicago, IL, USA), and an 11/12 explorer (Hu‐Friedy, Chicago, IL, USA). The protocol included the collection of intraoral photographic records and the following clinical parameters:

- Probing Depth (PD):
- Plaque Index (PI) (Silness and Löe 1964);
- Bleeding on Probing (BoP) (Ainamo and Bay 1975);
- Modified Gingival Index (mGI): adapted from the original Gingival Index (Löe and Silness 1963) by excluding the bleeding component and relying solely on visual assessment of the marginal gingiva;
- Suppuration on probing;
- Tooth mobility (Miller 1950);
- Gingival recessions (Rec) (Cairo et al. 2011);
- Clinical Attachment Level (CAL);
- Width of keratinized mucosa, measured as the distance between the gingival margin and the mucogingival junction (KMW);
- Width of Attached Gingiva, calculated as the difference between KMW and PD (AGW);
- Gingival phenotype, based on gingival thickness (GT) measured with the Colorvue Biotype probe (Figure 2) (Aslan et al. 2021).

Periodontal parameters were recorded to verify periodontal health according to the 2018 classification (Papapanou et al. 2018) and to characterize the site‐specific gingival–periodontal phenotype of individuals with ED.

### 2.4 Histological Analysis

A histological sample was obtained during a clinically indicated crown‐lengthening procedure required to facilitate fixed orthodontic appliance placement in a 16‐year‐old female patient with ED. A 5 mm gingival tissue fragment was collected from the buccal gingiva of tooth #4.6 and immediately fixed in 10% neutral buffered formalin for 24 h before paraffin embedding. Serial sections (3–4 μm thick) were obtained from the paraffin block. Sections were stained with hematoxylin and eosin (H&E) for general morphological assessment. For immunohistochemistry, additional sections were deparaffinized, rehydrated, and subjected to antigen retrieval. Endogenous peroxidase activity was quenched with hydrogen peroxide. The sections were then incubated with monoclonal antibodies against cytokeratin (CAM 5.2) and vimentin, followed by detection with a secondary antibody system. Visualization was achieved using a DAB chromogen, and nuclei were counterstained with hematoxylin. All immunohistochemical procedures were performed on a fully automated stainer (Ventana; Roche Diagnostics) to ensure standardized processing and reproducibility.

## 3 Results

The study included 11 patients (7 males and 4 females), with a mean age of 18.45 years (SD 4.29; range 16–30 years), all residing in Italy. All participants had a confirmed diagnosis of ED. In five cases, the diagnosis was supported by molecular testing: two patients presented mutations in the WNT gene, two in the EDA gene, and one in the EDAR gene. In the remaining six cases, three with anhidrotic ED and three with hypo‐hidrotic ED, the diagnosis had been established based on clinical findings and specific diagnostic tests, without genetic confirmation.

Ten patients exhibited varying degrees of oligodontia, while one patient was affected by hypodontia. A total of 22 deciduous and 92 permanent teeth were examined. Only two teeth, one primary and one permanent tooth anchored to an orthodontic arch, showed slight mobility. Teeth were classified based on the condition of the clinical crown, distinguishing between natural crowns, those restored with prosthetic materials, those modified by fillings, crowns with orthodontic appliances in place, or those undergoing other therapeutic interventions (Table 1).

**TABLE 1:** Distribution according to the clinical crown condition.

Clinical crown condition	*N*	%
No anomaly	58	51
Shape anomalies (a)	13	11
Fixed orthodontic appliance (b)	12	10
Fixed prosthesis on natural tooth	11	10
Composite restorations	5	5
Microdontia (c)	1	1
Co‐occurrence a–b	8	7
Co‐occurrence a–c	4	3
Co‐occurrence b–c	2	2

### 3.1 Clinical Periodontal Parameters

Regarding the clinical and inflammatory parameters, four patients had undergone one session of professional oral hygiene before enrolment. PDs were within the physiological range, with only two subjects presenting residual sites with PD greater than 3 mm. BoP was generally low, with the majority of sites scoring negative. The PI distribution indicated good oral hygiene, with most sites scoring 0 or 1 and only a minor proportion scoring 2 or 3. Similarly, mGI confirmed a satisfactory gingival condition, with a predominance of scores of 0 and very few sites scoring 2 or 3. No suppuration upon probing and tooth mobility was observed in any patient. Overall, all subjects exhibited a favorable periodontal and gingival status at the time of assessment. The distribution of periodontal parameters for each patient is summarized in Table 2.

**TABLE 2:** Individual periodontal parameters of enrolled patients.

Patient ID	No. of teeth	FMBS % (BoP+; BoP−)	Mean mGI (0–3)	No. of PI (0–1)	No. of PI (2–3)
1	4	0 (0; 24)	1.00	16	0
2	7	9.52 (4; 38)	1.68	25	3
3	2	9.09 (1; 11)	1.37	8	0
4	12	9.72 (7; 65)	0.39	39	9
5	13	5.13 (4; 74)	0.19	52	0
6	6	2.78 (1; 35)	0.75	24	0
7	13	0 (0; 78)	0.00	52	0
8	18	0 (0; 108)	0.00	72	0
9	12	6.95 (5; 67)	0.25	48	0
10	3	0 (0; 18)	0.00	12	0
11	24	3.33 (8; 136)	0.04	89	7

Abbreviations: FMBS, full‐mouth bleeding score; mGI, modified Gingival Index; PI, Plaque Index.

### 3.2 Periodontal Profile

The assessment of the width of attached gingiva and keratinized mucosa was conducted by sextant, rather than by tooth type, due to the known dental anomalies commonly observed in this population. Mean values for the width of attached gingiva were greater in the second sextant, as well as in the lingual regions of the fourth and sixth sextants (Table 3). Conversely, the narrowest measurements were recorded in the buccal aspect of the fourth sextant and the lingual aspect of the fifth sextant. The decreasing order of keratinized mucosa width across the sextants mirrored that of the attached gingiva, with the exception of the buccal aspect of the fifth sextant, which displayed higher values compared to the other mandibular regions (Table 4).

**TABLE 3:** Mean width of attached gingiva (SD) by sextant.

Sextant	Buccal	Lingual
First	3.07 (1.33)	—
Second	4.68 (2.42)	—
Third	4.00 (1.88)	—
Fourth	1.36 (1.29)	1.93 (1.54)
Fifth	1.67 (1.30)	1.50 (1.31)
Sixth	1.85 (1.21)	2.00 (1.41)

**TABLE 4:** Mean width of keratinized mucosa (SD) by sextant.

Sextant	Buccal	Lingual
First	4.78 (1.31)	—
Second	5.53 (2.28)	—
Third	5.17 (1.50)	—
Fourth	2.71 (1.20)	3.28 (1.53)
Fifth	3.50 (1.44)	2.37 (1.53)
Sixth	3.19 (1.11)	3.06 (1.53)

As for gingival thickness, thin gingiva was observed in 105 teeth. A medium‐thick phenotype was recorded in only four teeth, while five teeth exhibited a thick phenotype. The free gingiva often appeared swollen in comparison to the underlying mucosa and was partially or entirely lacking a defined gingival sulcus, especially around teeth adjacent to edentulous spaces, whether isolated or between teeth, where the interdental papilla was poorly developed or absent.

Due to the presence of restorations and structural enamel alterations, it was difficult to locate the cemento‐enamel junction in many cases, and as a result, clinical attachment level and gingival recession were not included in the analysis.

### 3.3 Histological Findings

**FIGURE 3:**
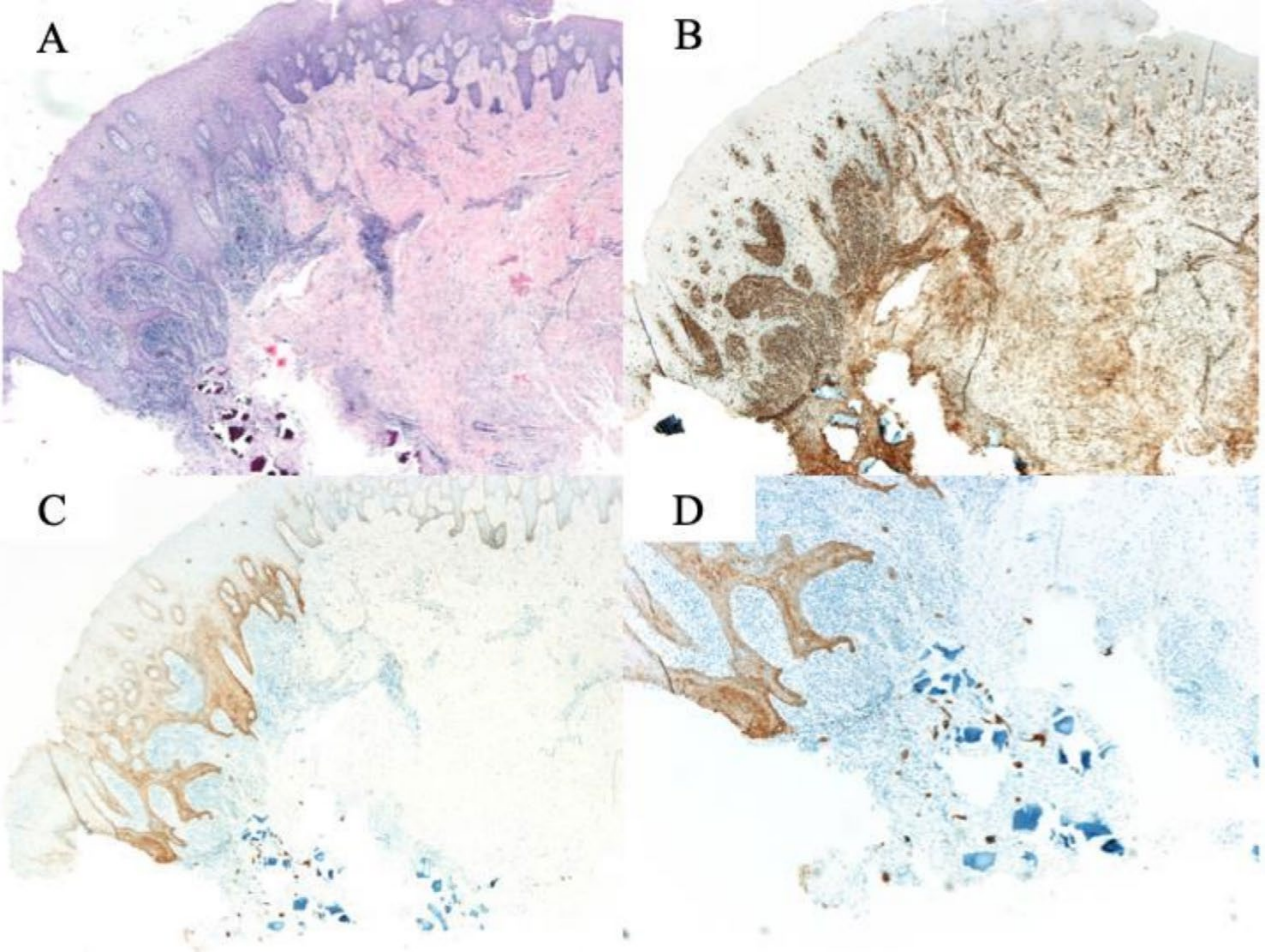
Histological and immunohistochemical features of gingival tissue from the patient with ectodermal dysplasia. (A) Hematoxylin–eosin staining (4×) shows irregular epithelial ridges and multiple epithelial buds extending into a poorly organized submucosa. (B) Vimentin immunostaining (4×) highlights numerous vimentin‐positive stromal fibroblasts beneath the epithelium, consistent with intense connective remodeling. (C) CAM 5.2 immunostaining (4×) demonstrates strong and diffuse cytokeratin expression outlining the epithelial compartment and the downgrowing epithelial buds. (D) At higher magnification (10×), CAM 5.2 immunostaining clearly delineates small cytokeratin‐positive epithelial nests embedded in a loose, myxoid stroma containing eosinophilic enamel matrix–like deposits, partially resembling the structure of an early dental follicle.

Histological examination of the gingival tissue from a single ED case revealed a markedly irregular epithelial surface, with elongated and uneven epithelial ridges giving rise to numerous epithelial buds extending into the underlying submucosa (Figure 3A). The connective tissue beneath the junctional epithelium appeared poorly organized, with loosely arranged collagen fibers and loss of the normal supra‐crestal orientation. Within this altered stroma, scattered epithelial nests and small foci of eosinophilic material resembling enamel matrix were observed, partially reproducing a dental follicle–like architecture.

Immunohistochemical analysis corroborated these morphological observations. Vimentin staining (Figure 3B) highlighted a dense population of vimentin‐positive stromal fibroblasts beneath the epithelial downgrowths, confirming an intense and disorganized remodeling activity within the submucosa. This pattern is consistent with an intrinsic alteration of the connective component, potentially underlying the clinical loss of gingival tone and the absence of surface stippling.

CAM 5.2 immunostaining (Figure 3C,D) demonstrated strong and diffuse cytokeratin expression sharply delineating the epithelial compartment. The cytokeratin labeling clearly outlined the rete ridges that deepened and branched into the submucosa, forming minute epithelial buds and nests surrounded by a loose, myxoid, fibroblast‐rich stroma. Within this stroma, small deposits of enamel matrix–like material were occasionally observed, often encircled by cytokeratin‐positive epithelial cells.

For comparison, a control gingival sample from an overall healthy subject, not suffering from ED and matched for age and gender, was selected from the histological archives of the Pathological Anatomy Institute (Figure S1). The analysis showed the normal histological organization of attached gingiva, characterized by a well‐stratified squamous epithelium with a continuous basement membrane and an underlying connective tissue composed of densely packed collagen bundles oriented along the typical supra‐crestal direction.

## 4 Discussion

The present study provides a descriptive analysis of gingival morphology and periodontal characteristics in a group of young individuals diagnosed with Ectodermal dysplasia (ED). Despite the limited sample size, several meaningful observations emerged that diverge from what is typically reported in the general population with respect to periodontal architecture.

Certain genetic conditions are explicitly included in the current classification of periodontal diseases, reflecting their recognized impact on periodontal health (Papapanou et al. 2018). ED, however, is not among them. Although the dental manifestations of this condition, such as anodontia or oligodontia, are well established, and the associated underdevelopment of the alveolar process often presents with a narrow, blade‐like profile, the focus has traditionally remained on restorative and prosthetic rehabilitation (Lamazza et al. 2009; Li et al. 2011; Pombo Castro et al. 2013). Whether the gingival and mucosal structures in these individuals follow conventional anatomical patterns or reveal deviations related to the broader ectodermal involvement remains an open question. This uncertainty served as the rationale for the present investigation.

The most consistent finding in our cohort was the predominance of a thin gingival phenotype, observed in the vast majority of examined sites. This stands in contrast to what has been reported in the general population, where thick phenotypes are often equally or more common, particularly in the anterior maxilla (Zweers et al. 2014; Kao et al. 2020). In the present study, medium or thick gingival tissue was recorded only in a few isolated molar sites, suggesting a widespread developmental alteration of soft tissues in ED. In many cases, the gingiva appeared exceptionally translucent, allowing for the visualization of the underlying capillary network, an aspect more commonly seen in elderly individuals due to age‐related epithelial thinning. In ED, this feature may reflect intrinsic histological deficiencies related to the ectodermal origin of the epithelium (Bergendal 2010).

Beyond phenotype, the gingival morphology itself deviated from standard anatomical expectations. The free gingiva often appeared edematous and lacked a clearly defined sulcus, particularly in areas adjacent to edentulous gaps, where the interdental papilla was frequently reduced or absent. These features do not conform to conventional descriptions of gingival architecture and may be influenced by the altered morphology of the dentition as well as by underlying connective tissue deficiencies. The marginal gingiva also exhibited pronounced flaccidity during probing, even in clinically healthy sites. This lack of firmness, regularly noted by the examiners, could suggest a deficit in epithelial‐mesenchymal cohesion, which has been previously documented in other ectodermally derived structures in ED (García‐Martín et al. 2013).

Measurements of attached gingiva and keratinized mucosa were generally within acceptable clinical ranges, but their distribution diverged from typical patterns reported in the general population (Ainamo and Löe 1966; Anand et al. 2022; Wennström 1987). While the anterior maxilla exhibited the expected greater widths, several mandibular regions, especially buccal and lingual aspects of premolars and canines, showed a marked reduction, and in some cases, a complete absence of attached gingiva. These patterns were accompanied by a modest degree of interindividual variability, which should be taken into account when interpreting the distribution of AGW and KMW values. Interestingly, some of the sites with the thinnest phenotype and reduced AGW did not show signs of gingival recession, suggesting that the limited soft tissue dimension was not necessarily a consequence of disease, but rather an expression of congenital mucosal architecture, likely linked to the ectodermal origin of the tissue. This interpretation is further supported by previous studies demonstrating that, in patients maintaining effective plaque control, the absence of an “adequate” zone of attached gingiva does not predispose to an increased risk of soft tissue recession (Wennström 1987; Lang and Löe 1972).

The present study provides a detailed histological and immunohistochemical characterization of gingival tissue from patients affected by ED, with a specific focus on epithelial architecture and stromal organization. The histological evaluation revealed a markedly irregular epithelial profile, with elongated rete ridges and multiple epithelial buds extending into a disorganized, fibroblast‐rich connective tissue. The subepithelial stroma lacked the dense, supracrestally oriented collagen bundles typical of normal attached gingiva, exhibiting instead a loose, myxoid texture interspersed with eosinophilic enamel matrix‐like deposits. These deposits were occasionally surrounded by cytokeratin‐positive epithelial cells, forming structures that closely resembled early dental follicle‐like arrangements. These features partially reproduced the architecture of a dental follicle and pointed toward a localized developmental disturbance within the gingival connective tissue. To strengthen the interpretation of these findings, the specimen was compared with gingival tissue from a healthy 16‐year‐old subject. This age‐matched comparison was essential to ensure reproducibility and to minimize potential confounding due to differences in gingival or dental developmental stage. The comparison confirmed that the observed disorganization of the connective architecture and epithelial downgrowths were not age‐related phenomena, but rather disease‐associated alterations.

The clinical assessment of gingival health in individuals with ED presents specific challenges, particularly when relying on conventional periodontal indices. In several cases, sites with low plaque accumulation and no overt signs of inflammation still returned elevated scores on the modified Gingival Index. This may be partially explained by the extreme thinness of the gingival epithelium, which allows the underlying capillary network to remain visibly prominent, mimicking the appearance of erythema. Similarly, bleeding on probing may not reliably indicate inflammatory activity, as the apparent clinical and histological fragility of soft tissues may make them more susceptible to mechanical disruption. The standard probing pressure used in periodontal examinations may inadvertently penetrate these soft tissues, resulting in false‐positive readings for both BoP and PD (Greenstein et al. 1981; Davenport et al. 1982).

The findings of this study underscore the importance of considering the soft‐tissue characteristics of individuals with ED in clinical decision‐making. Features such as a thin gingival phenotype, reduced attached gingiva, and increased tissue fragility might affect the stability of restorations, oral‐hygiene maintenance, and overall periodontal management.

This study has several limitations. The most significant is the limited sample size, which reflects both the rarity of the condition and the logistical challenges of recruiting individuals with ED. Moreover, the heterogeneity of the clinical and genetic diagnoses within the sample, although reflective of the real‐world ED population, makes it difficult to draw subtype‐specific conclusions. Another limitation was the difficulty in accurately identifying the cemento–enamel junction (CEJ) in many teeth. Altered crown morphology, microdontia, enamel defects, and the presence of restorations often obscured the CEJ, introducing potential variability in measurements such as AGW, KMW, and clinical attachment level. This limitation may have affected the reliability of the linear measurements and must be considered when interpreting the clinical comparisons. Additionally, gingival thickness was assessed using a color‐coded probe rather than ultrasound or transgingival methods, which may have introduced variability in classification. Despite these constraints, the study provides preliminary insights into the soft‐tissue anatomy of a rarely studied population and may serve as a foundation for future investigations involving more refined techniques and broader cohorts.

## 5 Conclusions

The observed deviations in gingival phenotype, tissue distribution, and mechanical response suggest that the oral mucosa in ED may reflect the broader ectodermal and mesenchymal involvement of the syndrome. These findings emphasize the need for heightened clinical awareness, careful diagnostic interpretation, and tailored treatment approaches. Future research with larger cohorts, histological analysis, and genetic characterization may further clarify the periodontal implications of ED and the contribution of genotype to gingival phenotype expression.

## Author Contributions

**Marco Montevecchi:** conceptualization, supervision, writing – review and editing, methodology. **Margherita Giorgia Liguori:** visualization, writing – original draft, data curation. **Ilaria Santini:** conceptualization, data curation, investigation, writing – original draft. **Nicola Marco Sforza:** validation, conceptualization, writing – review and editing. **Giulia Querzoli:** data curation, investigation, writing – review and editing. **Leoluca Valeriani:** conceptualization, supervision, data curation, writing – original draft.

## Funding

The authors have nothing to report.

## Conflicts of Interest

The authors declare no conflicts of interest.

## Supporting information


**Figure S1:** Control gingival sample showing the normal architecture of attached gingiva, with a well‐stratified squamous epithelium and a dense connective tissue composed of collagen bundles oriented in the typical supracrestal direction.

## Data Availability

The data that support the findings of this study are available from the corresponding author upon reasonable request.
