# Supplementary material for: Periodontal Architecture in Ectodermal Dysplasia: An Observational Clinical and Histological Study
Source: Oral Dis. 2026 Jan 8;32(5):1451–9. doi: 10.1111/odi.70196 (PMC13365010; doi:10.1111/odi.70196)
Supplement: Supplementary file 1 — Figure S1: Control gingival sample showing the normal architecture of attached gingiva, with a well‐stratified squamous epithelium and a dense connective tissue composed of collagen bundles oriented in the typical supracrestal direction. [file ODI-32-1451-s001.docx]

**SUPPLEMENTARY FILE:**

**Supplementary Figure 1.** Control gingival sample showing the normal architecture of attached gingiva, with a well-stratified squamous epithelium and a dense connective tissue composed of collagen bundles oriented in the typical supracrestal direction.

**
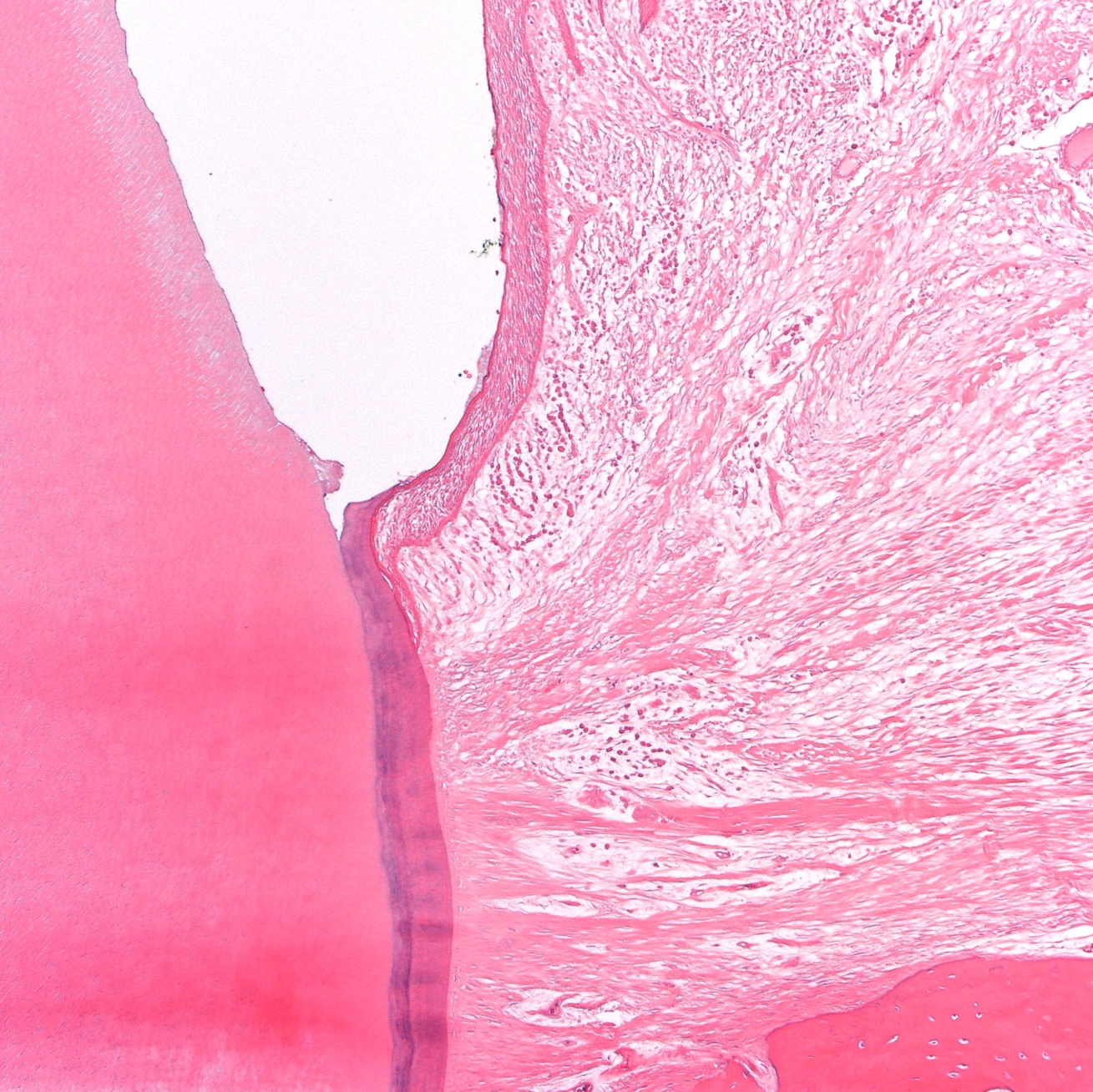
**
